# Recent findings on organizational unlearning and intentional forgetting research (2019–2022)

**DOI:** 10.3389/fpsyg.2023.1160173

**Published:** 2023-07-27

**Authors:** Annette Kluge

**Affiliations:** Chair of Work, Organizational, and Business Psychology, Faculty of Psychology, Ruhr University Bochum, Bochum, Germany

**Keywords:** innovation, relearning, knowledge, readiness, beliefs, routines, organizational memory

## Abstract

This mini review aims at summarizing the current state-of-the-art of empirical unlearning and intentional forgetting (U/IF) research at the individual, team, and organizational level. It adds to an earlier review and incorporates 31 recent studies from 2019 to 2022. The review reveals that predictors based on the organization’s adaptation context (e.g., competitive intensity), organization level (e.g., leadership exploration activities), individual task-related (e.g., features of the routines changed), and person-related level (e.g., cognitive control strategies) variables relate to process variables, such as the type of U/IF, the U/IF content (e.g., success beliefs or failure beliefs), and information processing variables (e.g., team information processing). The outcome variables are at the organizational level (e.g., cross-boundary innovation), team level performance level, the individual task performance level (e.g., errors), and person-related level (e.g., self-esteem). The analyzed studies at the team and organizational levels preferred cross-sectional study designs or in-depth qualitative methods, which severely limits the possibility of making causal statements. In contrast, at the individual-level studies use longitudinal designs as well to make temporal aspects of U/IF visible. But these individual level results are limited in terms of their generalizability to other levels. Even though all studies make valuable contribution to the understanding of antecedents and outcomes of U/IF, the temporal and process-related aspects of how U/IF unfolds at the different levels and subsequent options for its deliberate facilitation remain empirically little elaborated. It is proposed that in addition to studying the antecedents and consequences of U/IF in cross sectional designs, the topic needs more longitudinal designs to capture the nature of the U/IF processes in organizations.

## 1. Introduction

Why is this review needed? Individual, team, and organizational memories shape present and future choices, behaviors, and strategies (Rowlinson et al., 2010; Foroughi et al., 2020). This review aims to summarize empirical evidence on the organizational memory processes of organizational unlearning and intentional forgetting (U/IF). Memory in organizations has several “bins”: individual memories, team memories, organizational routines and practices, and digital storage bins (Walsh and Ungson, 1991). The purpose of the present mini review is to summarize the recently added *empirical* results from 2019 to 2022. The previous reviews by Klammer and Güldenberg (2019) and the reviews by Sharma and Lenka (2022a,b) already covered the theoretical state of the art and the emergence of the concept of unlearning and forgetting, the paper by Kluge and Gronau (2018) and Kluge et al. (2019) covers the empirical state of the art up to the year 2018. The present mini review is supposed to inspire and support the recently increasing efforts of implementing additional empirical research in the field of U/IF to catch the momentum.

This review is needed because the gap between theoretical assumptions and the number of articles on U/IF models is growing much faster than the number of empirical papers that attempt to test at least some of the model assumptions (Kluge et al., 2019). From a theoretical-conceptual perspective, there is certainly no lack of hypotheses, propositions, and models on the topic at present, but there is a lack of serious efforts to test these manifold assumptions in a consistent and interrelated manner. From an empirical perspective, there is currently a lack of interrelated approaches to empirical questions and the use of measurement instruments. That leads to construct confusion that creates difficulty in building a cohesive body of scientific literature by not finding the same language to talk about the challenges of describing, understanding, and supporting U/IF in organizations.

What happened so far in the unlearning and intentional forgetting literature? In recent decades, U/IF, as an organizational concept, has received attention in the discussion on the prerequisites for learning in organizations, innovation, management of change, and new product development (Kluge and Gronau, 2018; Sharma and Lenka, 2022b). The concepts of U/IF have attracted researchers from a wide spectrum of interests, such as innovation, development, information systems, knowledge management, and human resources (Durst et al., 2020). In the U/IF literature, the individual, group, and organizational levels of analysis, as well as elements that should be replaced, such as knowledge, routines, habits, mental models, or behaviors, have been identified (Kim and Park, 2022; Sharma and Lenka, 2022b). For example, U/IF has entered the scholarly discussion on the dynamic capabilities of organizations (Khin Khin Oo and Rakthin, 2022), their absorptive capacity as the ability to acquire, assimilate, transform, and exploit new external knowledge to achieve competitive advantages and superior performance (Zahra and George, 2002), and the context of organizational resilience, knowledge creation and integration capabilities, team information processes, and knowledge sharing. Recent technical developments, especially in combination with machine learning, have led to new topics, such as U/IF supported by a socio-digital system design for augmenting human cognitive performance in technical systems (Kluge and Gronau, 2018; Hertel et al., 2019; Thim et al., 2019) and the use and development of assisting technology-aided IF digital solutions (Ellwart et al., 2020).

As the theoretical distinction between unlearning and intentional forgetting is not well defined, both concepts are combined in the present review. Both concepts include the process of “letting go” of once-useful mindsets (O'Reilly, 2018, p. 19) and acquired behaviors that were effective in the past but now limit success. According to previous reviews (e.g., Tsang and Zahra, 2008; Kluge and Gronau, 2018; Klammer and Güldenberg, 2019; Kluge et al., 2019; Sharma and Lenka, 2022a; Kim and Park, 2022), *U*/IF in organizations involves attitudes and processes that deliberately impede the recall of certain organizational memory items from organizational storage bins, such as individual or team memories or routines and practices, to adapt to the changing affordances in the (market) environment.

As mentioned above, the need for an updated review of empirical results is derived from the ongoing imbalance between the (small) number of empirical studies compared to the (high) number of conceptual papers (Kluge and Gronau, 2018; Kluge et al., 2019; Durst et al., 2020; Sharma and Lenka, 2022a,b). Additionally, the small number of empirical studies so far have often used the same research methods and are mainly static and cross-sectional (Durst et al., 2020). The statement by Brook et al. (2016, p. 383) that “much of the unlearning literature is conceptual, speculative and lacking in empirical data” still applies (Klammer and Güldenberg, 2019; Becker and Bish, 2021).

As the research gap is inherently obvious due to the small number of empirical studies in comparison to the number of theoretical models and concepts, the present mini review aims at encouraging researchers to build on existing results, discuss the existing research methods and to compared empirical approaches with a variety of research strategies, to add findings from different, e.g., national or business contexts and thereby to jointly built a comprehensive understanding of the antecedents, processes and results of unlearning and forgetting in “real life.”

Otherwise, it may be feared that the hitherto fragmented empirical landscape and the associated inconsistent field of findings will weaken the persuasiveness and thus the use of the concept, and that researchers and practitioners will turn away from U/IF in frustration.

## 2. Review process and results

### 2.1. The review process

This review was conducted based on the guidelines of Tranfield et al. (2003) on how to undertake a systematic review by searching leading electronic databases, including peer-reviewed publications, conference proceedings, and internet sources listed in Google Scholar, PsycArticles, PsyINFO, and Psyndex (via EBSCO). A search was conducted using the terms “organizational unlearning + study,” “organizational unlearning + study,” “organizational forgetting + study” and “organizational forgetting + study,” “organizational unlearning + empirical,” “organizational unlearning + empirical,” “organizational forgetting + empirical,” and “organizational forgetting + empirical.” A total of 31 scientifically sound empirical studies in English, published between 2019 and 2022 (including online first articles) in scholarly and peer-reviewed journals, peer-reviewed conference full papers, and one book, were identified as having a direct relation to the review objectives (13 at the individual level, three at the team level, and 15 at the organizational level). Scientifically sound means that the studies included adhere to the ethical standards (e.g., participants were asked for informed consent, voluntary participation) and were published in peer-reviewed journals or conference proceedings, that checked for standards of conducting and reporting empirical research (e.g., reporting the selection of the sample, response rates, using valid and reliable instruments, using appropriate statistical analysis).

The selected conference proceedings were included as scholars in the field of U/IF from business information systems publish in conference proceedings (and less often in journals). Only those studies with U/IF in their titles that referred to an intentional and deliberate process to actively adapt to a changed environment or task were included. Studies that included accidental forgetting were excluded.

### 2.2. Results

As the empirical state of the art is in the center of the present mini review, the studies included are presented with an emphasis on the central empirical research question, the research strategy (e.g., qualitative, quantitative, cross sectional—longitudinal), the sample, the level of analysis (individual, team, organization) and the main findings.

The levels of analysis (individual, team, organizational) were chosen as they represent shared assumptions in organizational psychology, knowledge management, and organizational behavior about on “where” organizational memory storage bins and processes are located and “where” unlearning processes unfold (Huber, 1991; Walsh and Ungson, 1991; Crossan et al., 1999; Becker, 2005; Akgün et al., 2006; Argote, 2013; Zhao et al., 2013; Akhshik, 2014). The studies found addressed mainly one level only and can consequentially sorted into one of the levels based on the definition and description of the used sample (individuals, teams, or organizations).

#### 2.2.1. U/IF at the individual level

##### 2.2.1.1. Person-related aspect of U/IF

Niessen et al. (2019) conducted an exploratory qualitative study with 65 working participants to investigate the functions of U/IF in a work context. They identified emotion regulation, preservation of self-image, maintenance of social relationships, knowledge acquisition, goal attainment, and maintaining attentional control as functions.

In an experimental longitudinal laboratory setting, Niessen and Lang (2021) investigated the role of *cognitive control strategies* to support U/IF in air traffic control task adaptation in two experimental studies (*N* = 66 and *N* = 105). The participants first learned and performed an initial version of the task, received different instructions for control strategies (either to deliberately forget old rules, remember the old rules, or simply learn the new rules), performed an altered version of the task with new execution rules, and finally worked on a memory test. The instruction to intentionally forget best supported the participants’ performance in applying the new rules.

In a cross-sectional field study, Kmieciak (2020) investigated employees’ innovative work behavior (idea generation and realization) as a result of individual unlearning and affected by *critical reflection*. Critical (premise) reflection is perceived as a higher-order, active, and purposeful process of investigating the justifications for one’s beliefs. Kmieciak (2020) used survey data (unlearning scale) from 252 Polish employees (69 managers). Critical reflection showed both direct and indirect effects on idea generation and realization through individual unlearning. Problem-solving demands correlated with critical reflection. While the subsample of employees’ results showed positive correlations between unlearning and innovative work behavior, correlations between critical reflection and innovative work behaviors became apparent in the manager subsample.

Comparably, Matsuo (2021) investigated the effects of goal orientation (learning goal orientation and performance goal orientation) on individual U/IF through reflection and *critical reflection* using survey data from 271 employees of Japanese organizations. Like Kmieciak (2020), Matsuo (2021) found that critical reflection had a positive direct effect on unlearning, whereas reflection alone had a complete indirect effect on U/IF through critical reflection. Goal orientation had positive direct effects on both reflection and critical reflection. Matsuo (2021) further investigated the effects of critical reflection on U/IF and work engagement through a survey of 301 employees. The results showed that reflection facilitates U/IF and work engagement through critical reflection.^1^ Haase et al. (2020) used a similar lab-based production context and showed in a pre-post-test design with 41 participants within a group design that the participants’ retentivity (as a facet of intelligence) largely explained variance in individual differences in intentional forgetting performance.

In a combination of an experimental laboratory study and a longitudinal field study, Göbel and Niessen (2021) assessed 143 employees’ individual abilities to suppress thoughts, followed by a 5-day experience sampling study in a work context. Multi-level analyses showed that individuals with lower suppression abilities experienced higher negative affect and lower self-esteem when they tried to suppress intrusive thoughts to support U/IF. In contrast, individuals with higher suppression abilities did not. Niessen et al. (2020) also conducted an experience sampling study combined with a laboratory task to assess the ability to suppress the unwanted thoughts of 158 workers. The workers engaged more often and more intensively in thought control activities at a moderate level of time pressure but only when they had a higher general ability to suppress unwanted thoughts. For workers with a lower ability to suppress unwanted thoughts, increasing time pressure was negatively correlated with thought control activities, even at very low levels of time pressure.

##### 2.2.1.2. Task related aspects and technology assisted U/IF

Brooks et al. (2022) conducted a qualitative study (i.e., observation, focus groups, and semi-structured interviews) of learning in the United Kingdom Fire and Rescue Service involving 12 fire stations, 44 firefighters, and 14 senior managers. The intention was to understand the social aspects of unlearning, for example, in the people involved as active agents rather than passive recipients or discarders of knowledge. *Practices or procedures* that were outdated, rarely consulted, or used were easy to unlearn. Knowledge and skills that were no longer relevant to the current practice were not completely unlearned but remained as interesting memories or amusing anecdotes. Firefighters needed to trust the effectiveness of any new practices or the credibility of new knowledge to consent to unlearning the old practices and replacing them with new ones.

Cegarra-Navarro et al. (2021) conducted an empirical (cross-sectional) study with 122 airline travelers on U/IF in the context of COVID-19-related changes in traveling and defensive *routines*. U/IF was negatively related to defensive routines.

In an experimental laboratory study using a simulated sales planning task supported by a computer-based decision and U/IF-support system (DSS), Hertel et al. (2019) (*N* = 90) found that the availability of DSS triggered the forgetting of decision-related background information, which in turn increased users’ *mental resources* for additional tasks, *decision quality, and well-being*. Moreover, *trust* in the system was found to be a relevant predictor of “letting go.” Meeßen et al. (2020) used the same scenario and replicated the findings with 200 participants in an experimental design, which manipulated the level of trustworthiness of the decision support system. Trust was confirmed to significantly enhance intentional forgetting, performance, and well-being.

Similarly, Roling et al. (2023) used an experimental pre-posttest study (*N* = 16 workers in a production line) to discover the differences in U/IF dependent on a continuous or episodic change of a production routine. In continuous change conditions in which changes in specific production steps occurred stepwise during multiple production processes, U/IF performance depended on the *kind of routine changes*: actions that were newly introduced and actions that needed to be omitted were more difficult to forget than changes in the way a specific step needed to be executed. Additionally, the participants made the same “forgetting errors” repeatedly and subsequently after an action changed and were maintained over time.

#### 2.2.2. U/IF at the team level

##### 2.2.2.1. U/IF and links to risk aversion and an error forgiving climate

In a holistic multiple-case study on new product development (NPD) teams, Klammer and Güldenberg (2020) conducted 30 semi-structured interviews with NPD team members using additional archival data. They found that daily routines and *risk aversion* were antecedents of inability and resistance to U/IF. By contrast, raising awareness, providing temporal and spatial freedom, and facilitating an *error-forgiving climate* support U/IF through a more entrepreneurial, error-forgiving, or open-minded organizational culture that enables teams to break free from obsolete routines, patterns, mental models, or perceptions.

##### 2.2.2.2. U/IF and team information processing and experimenting

In a cross-sectional survey-study, Amaya et al. (2022) found that team U/IF was an important precondition for success in NPDs, as they needed to handle vast amounts of information processed to generate new ideas. Based on the data of 255 NPD team members from 80 firms, U/IF showed its effect on *teams’ information processing*, which subsequently led to NPD success (Amaya et al., 2022). The relationship between team unlearning and NPD success was fully mediated by team information processing.

Similarly, Matsuo (2021) investigated managers’ *exploration activities*, including experimenting with new business approaches, and reconsidering existing beliefs and decisions, in critical reflection and U/IF among subordinates (115 employees in 23 teams) at the team level. The results showed that managers’ exploration activities promoted individual unlearning through the mediating effects of learning goal orientation and reflection.

#### 2.2.3. U/IF at the organizational level

##### 2.2.3.1. Environmental and organizational characteristics affecting U/IF

Concerning the effect *of organizational culture* based on the Organizational Culture Assessment Inventory, OCAI instrument (i.e., market, clan, adhocracy, and hierarchy culture) on U/IF, Leal-Rodríguez et al. (2019) investigated the relationship between the OCAI facets, U/IF, and innovation. The results were based on data from 145 senior executives from companies, and they showed that market culture had a positive effect on U/IF and innovation. Clan culture exerted a negative effect on U/IF, while its link to innovation was not significant. A direct positive relationship was found between U/IF and firm innovativeness.

In the construction sector, Wong et al. (2021) investigated U/IF using scales of Akgün et al. (2006, 2007a,b), with 104 respondents in the context of contractors’ readiness to use prefabricated products for any building parts for onsite installation. They found that *organizational readiness* (an antecedent of practice change) was supported by U/IF, with a stronger effect of U/IF on routines than on beliefs. By contrast, the presence of gossip and counterfactual knowledge decreased the likelihood of organizational readiness for using prefabricated building parts.

In the context of *competitive intensity*, which is the degree to which a firm faces competition in its market and a firm’s products can be quickly replaced by those of other competitors, a cross-sectional survey study by Lyu et al. (2022) with 242 firms illustrated that *competitive intensity* is positively associated with knowledge integration and U/IF. However, firm size strengthened the relationship between competitive intensity and knowledge integration and weakened the relationship between competitive intensity and organizational unlearning.

##### 2.2.3.2. U/IF linked to organizational knowledge and innovation management, learning, and relearning

The relationship between U/IF, *knowledge management* and organizational outcomes was addressed in the study by Delshab et al. (2021), in which 316 members of the boards of directors of community sports clubs participated in the cross-sectional survey. U/IF showed positive impact on knowledge management and organizational outcomes. Furthermore, knowledge management activities mediated the relationship between the U/IF and organizational outcomes.

In the context of sustainable markets, Zhao et al. (2022) explored how U/IF *affects inclusive innovation* through supply chain green learning and the moderating role of green control ambidexterity using survey data from 217 firms. Inclusive innovation means providing support to low-income groups to participate in innovation activities equally and share innovation achievements (Zhao et al., 2022) in a small town and a vast rural market. Zhao et al. (2022) divided organizational U/IF into (a) non-environmental forgetting and (b) environmental change based on the perspective of *knowledge management* and changes in conventions and beliefs. Supply chain green learning mediated the effects of U/IF and environmental change on symbolic and substantive inclusive innovations.

Ayduğ and Ağaoğlu (2023) designed a similar cross-sectional survey based on data from 524 participants from the education sector. The study used self-developed scales on organizational learning, (intentional and accidental) organizational forgetting scales, and innovation management. The results showed a positive relationship between organizational learning and U/IF and between U/IF and *innovation management*. U/IF had a partial mediating effect on the relationship between organizational learning and innovation management.

To study the relationship between U/IF and breakthrough and to cross the boundary from the original limits, Qu et al. (2022) collected data from 353 middle and senior managers from entrepreneurial enterprises in China. U/IF was found to have a significantly positive effect on *cross-boundary innovation*. Binary knowledge sharing (exploitation and exploration knowledge sharing) played a mediating role in the relationship between organizational forgetting and cross-boundary innovation. As the mediating effect of exploratory knowledge sharing was more robust than exploitative knowledge sharing, the authors concluded that “abandon the old” and “discipline the new” in U/IF could continuously promote positive organizational development.

##### 2.2.3.3. U/IF and its link to organizational capabilities

With an emphasis on *social media strategic capability*, which is the ability to acquire and integrate information from social media into its knowledge base and the optimization of in- and outflow of knowledge in alignment with its strategic directions, Zhang and Zhu (2021) investigated the mediating role of U/IF and top management team diversity on disruptive innovation based on a sample of 198 manufacturing companies. They revealed that U/IF mediated the relationship between social media strategic capability and disruptive innovation. In addition, the effect was amplified (a) in companies with heterogeneous top management teams, and (b) was increased in dynamic markets and regulatory environments but was (c) weakened in dynamic technological environments.

Addressing the setting of cross-border mergers and acquisitions (M&As), Xi et al. (2020) explored the relationships between U/IF and knowledge transfer from a routine-based view with 178 samples from multinational corporations that experienced cross-border M&As. Results revealed that organizational *knowledge integration capability* provides an important connection between U/IF and interorganizational knowledge transfer. Practically spoken, U/IF supports discarding useless routines and integrating useful ones.

To investigate the relationship between U/IF (updating routines and knowledge) and relearning jointly facilitating strategic flexibility, Zhao and Wang (2020) used survey data from 194 firms and found that U/IF promotes (fully mediates) organizational relearning, thereby improving *strategic flexibility*.

Raisal et al. (2019) explored the effects of *knowledge creation capability*, U/IF, and absorptive capacity on firms’ innovative performance. The data were collected from 194 small- and medium-sized enterprises (SMEs; CEOs). In their study, knowledge creation capability positively influenced the correlation between U/IF on firms’ innovative performance. Additionally, *absorptive capacity* mediated the relationship between knowledge creation capability, U/IF, and innovation performance.

Orth and Schuldis (2021) used online survey data from employees of German and Austrian organizations based on a capability approach to understand U/IF during the COVID-19 crisis in terms of learning and resilience. They found no moderating effect of unlearning on the relationship between learning and organizational resilience.

##### 2.2.3.4. U/IF’s content and the nature of forgetting

Grisold et al. (2020) examined the facilitators of U/IF from a consultant perspective. They interviewed 24 change consultants and found differences between *open-ended U/IF* (organizational knowledge is intentionally discarded, but the outcomes of the change process are not known) and *goal-directed U/IF* (organizations implement specific knowledge structures incompatible with established ones). Open-ended U/IF requires breaking patterns, creating space for experimentation, ensuring (transparent) communication, providing time, and encouraging failure. Conversely, goal-directed U/IF requires splitting a change process into steps while providing actors with a clear idea and guidelines of what they should unlearn and forget while reducing the effect of outdated knowledge (e.g., impediment of reinforcement of outdated knowledge).

Based on a simulation study, Martignoni and Keil (2021) investigated the differences in outcome between U/IF of what had worked in the past (*success beliefs*, which embody what an organization believes to be related to positive outcomes) and unlearning of what did not work (*failure beliefs* or choices with negative consequences). They found positive short-term effects of U/IF on success beliefs and long-term effects of U/IF on failure beliefs. Organizations gain more from unlearning failure beliefs because organizational failure experiences generally exceed the number of success experiences. Both types of unlearning are superior to not unlearning in a wide range of different extents of environmental changes.

#### 2.2.4. U/IF at the interorganizational level

In the period of 2019 to 2022, there was no empirical paper published on the interorganizational level that met the inclusion criteria (see Review process section), but one paper addressed the industrial district level. At the industrial district (ID) level (i.e., a population of local specialized firms—micro-to-medium-sized and independent—contributing directly or indirectly to the localized main industry), Bellandi et al. (2018) conducted three case studies in the United Kingdom and Italy. The results showed that IDs successfully applied U/IF because of newly introduced (European Union) regulations (e.g., pollution) and new materials (e.g., new fiber) but were not able to adapt with technology against global competition. Additionally, after successful U/IF, some IDs were not able to reuse unlearned knowledge, which suddenly became required (e.g., after commercial restrictions from Russia in 2014).

## 3. Summary and conclusion

### 3.1. Integration of findings

**Figure 1:**
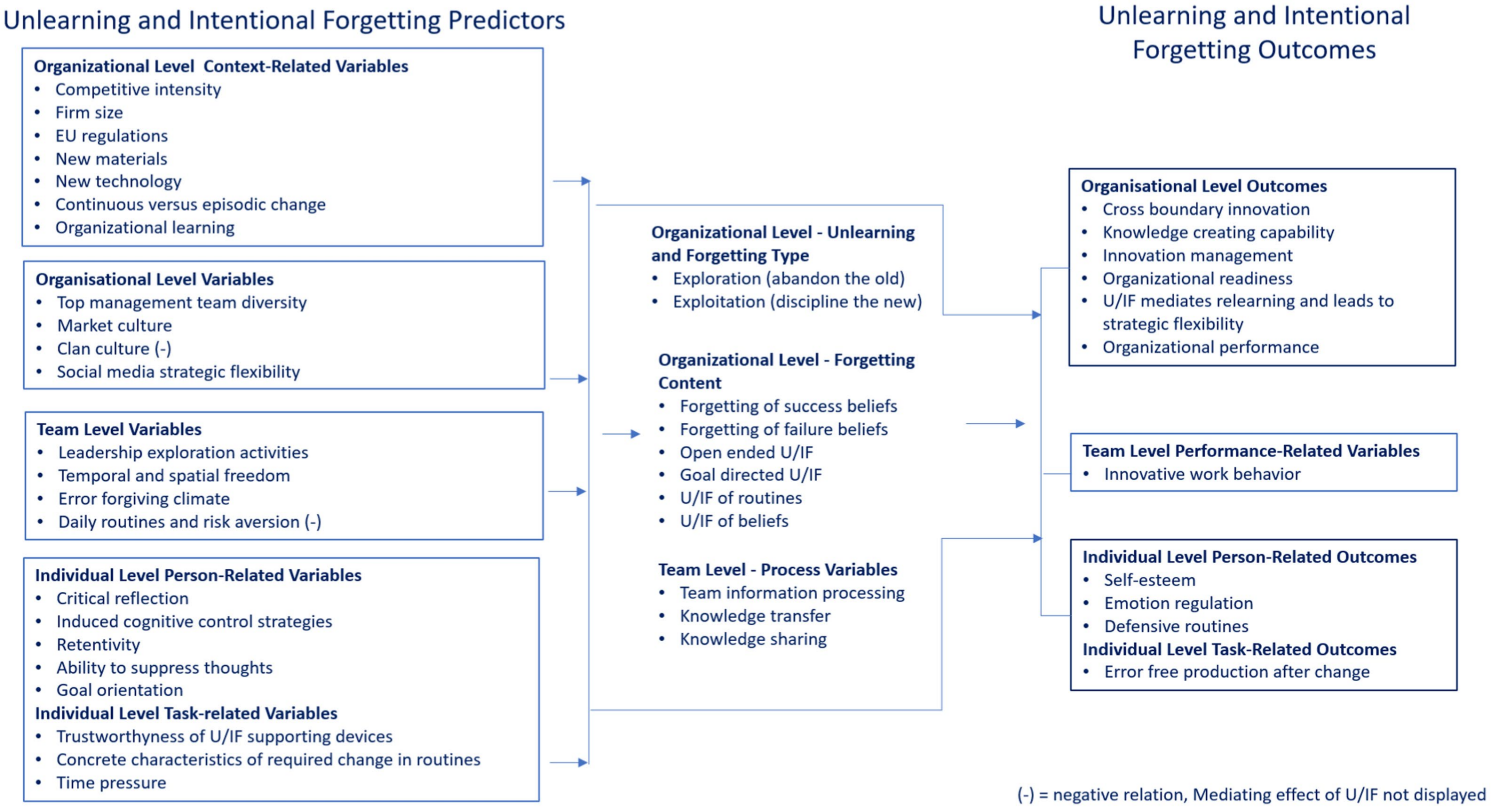
Predictors and outcomes of unlearning and intentional forgetting (U/IF) and U/IF-related process variables found in the empirical studies analyzed (no mediating effects shown).

Figure 1 shows the integration of the review findings clustered into predictors of U/IF, process aspects, and U/IF outcomes on the individual, team, and organizational level. Based on the findings, the individual level variables are divided into person-related and task related predictors and outcomes. To provide an overview and to illustrate the general picture of main effects and results, mediating effects are not displayed.

The results revealed predictors based on the following:

- Organizational level context related variables (e.g., competitive intensity, EU regulations, available new materials and technologies, firm size, and episodic vs. continuous change).
- Organizational level variables (e.g., heterogeneous top management teams, cultural aspects, providing guidelines of what should be unlearned and forgotten, reducing the influence of outdated knowledge, and impeding the reinforcement of outdated knowledge and routines).
- Team level variables (e.g., leadership exploration activities, breaking patterns, creating space for experimentation, providing time, encouraging failure, and risk aversion).
- Individual level person-related variables (e.g., critical reflection, cognitive control strategies, retentivity, ability to suppress thoughts, goal orientation, and defensive routines).
- Individual level task-related variables (e.g., features of new routines and kinds of elements to be forgotten compared with the previous one, time pressure).

The studies also addressed the following variables:

- Type of U/IF (e.g., abandon the old vs. discipline the new)
- Forgetting content (e.g., success beliefs or failure beliefs, open-ended vs. goal-oriented U/IF)
- Processing variables (e.g., during team information processing when generating new ideas)

The outcome variables were found at the following levels:

- Organizational level (e.g., cross-boundary innovation, readiness, positive organizational development, knowledge creation capability in innovative and innovation performance, knowledge management, and organizational performance), with diverse temporal dynamics of unlearning success or failure beliefs.
- Team level (e.g., innovative team behavior).
- Individual level task related (e.g., performance errors) and person-related variables (e.g., self-esteem).

Several mediating effects were reported (not displayed in Figure 1). U/IF had a partial mediating effect on the relationship between organizational learning and innovation management, fully mediated relearning and thereby improving strategic flexibility, and mediated the relationship between social media strategic capability and disruptive innovation. The relationship between U/IF and organizational outcomes in several studies was dependent on the quality of knowledge management activities (e.g., knowledge sharing, transfer, and integration).

### 3.2. Implications for further research

The message of this review is, that at the end of 2022 we had already some insights on what prompts, supports and enhances U/IF on individual, team, and organizational levels. Additionally, we know more about which variables are affected in terms of criteria and dependent variables. What remains less well elaborated is the process of becoming aware of U/IF requirements and how this awareness leads to actions on starting a U/IF process.

To give some examples: Findings on antecedents and consequences of U/IF suggest, that U/IF is facilitated and supported by environmental and management factors as well as elements of the task and individual dispositions. The findings related to the outcomes of U/IF show that U/IF supports characteristics of the organization also referred to as dynamic capabilities. As the U/IF research share some proposition with related concepts such as learning, innovation and knowledge management, further research should clarify in how far U/IF is distinct from or overlapping with organizational learning, innovation processes and knowledge sharing, e.g., to understand its unique contribution to an organization’s dynamic capabilities and the distinct underlying processes.

Additionally, we need to better understand the “gate” through which the U/IF requirements enter the organization. The gate could be a department (e.g., marketing that receives and interprets customer feedback and reviews social media discussions or legal services who report about changes in, e.g., EU regulations?) or a single person (a manager responsible for strategic management or the Chief Innovation manager?). Second, we need a deeper understanding of how the discovery of U/IF requirements diffuse through the organizational structures if there are no U/IF processes implemented so far? How is U/IF in organizations operationalized in terms of process-steps of a dynamic capability? Third, we know about the barriers to organizational learning, will we also find barriers to U/IF? Can we proactively enhance U/IF processes in teams and organizations? Are there facilitation techniques that are more powerful compared to others? Do effect sizes of facilitation techniques depend on the nature of the organization or the market environment in which it operates?

Even though the reviewed studies make an important contribution to the understanding of antecedents and outcomes of U/IF, the temporal aspects of how U/IF unfolds at the different levels and subsequently options for its deliberate facilitation appear empirically little elaborated. That means that from a research strategies perspective, the empirical investigation of underlying processes of U/IF (and not U/IF as a result) and the possible impacts of its deliberate facilitation are underinvestigated. Additionally, the models that are tested are not very well linked to previously established conceptual or theoretical models on U/IF. Further research is needed that systematically links (earlier) theoretical proposition, e.g., by Tsang and Zahra (2008) or Martin de Holan et al. (2004) on U/IF with corresponding research strategies. That means, so far theory progression and implemented research strategies are not well connected. There seem to be several “extremes”: cross sectional studies covering many organizations on a very high level of abstraction (e.g., Zhang and Zhu, 2021), in-depth qualitative case studies and observations of processes of one particular organization (e.g., Brooks et al., 2022), or laboratory studies that make use of controlled longitudinal designs with individuals. A balance between strategies, e.g., such as longitudinal (mixed-method) field studies, that allow for causal conclusion in combination with strategies that can capture the dynamics and the temporal dimensions of U/IF are desirable. Cross-sectional research designs face challenges of internal validity and allow not for concluding causal relationships. To further improve the understanding of the U/IF, more longitudinal-designs and mixed methods designs in teams and organizational “real world” contexts are necessary to produce insights that integrate criteria for high internal and external validity: e.g., a longitudinal study that accompanies an U/IF process that uses online experience sampling techniques (e.g., short questionnaires distributed at several measurement points during a 2 years U/IF process) that capture the dynamics of the U/IF process in combination of interview studies with responsible decision makers, innovation managers or management board members and focus group discussion with the U/IF and Change Management Team sheds light into the sequence of events, their dynamics, ups and downs, and the acceleration and deceleration of U/IF processes. Such a research strategy goes beyond the mere statement at hindsight that U/IF happened or not, as some of the frequently used instruments measure.

In sum, it is a positive sign that the number of empirical studies of U/IF has increased, but for future theory building and empirical evidence developments, research at different levels needs to be more systematically related and integrated to support the progression of the U/IF field.

The world is full of global challenges and crisis—contributing to the understanding and shaping of U/IF processes in organizations as an important coping strategy to face these future challenges might make a relevant difference if we will succeed.

## Author contributions

AK conducted the literature search, analyzed the papers, and wrote, edited, and revised the paper.

## Funding

This study was supported by the German Research Foundation (Deutsche Forschungsgemeinschaft), with grant number KL2207/5-2 and KL2207/6-2.

## Conflict of interest

The author declares that the research was conducted in the absence of any commercial or financial relationships that could be construed as a potential conflict of interest.

## Publisher’s note

All claims expressed in this article are solely those of the authors and do not necessarily represent those of their affiliated organizations, or those of the publisher, the editors and the reviewers. Any product that may be evaluated in this article, or claim that may be made by its manufacturer, is not guaranteed or endorsed by the publisher.

## Footnotes

^1^In an additional study by Matsuo (2021), the U/IF upon promotion from senior manager to executive officer was qualitatively examined using interview data from 46 executive officers in medium- and large-sized Japanese firms, which was already included in review of Kluge and Gronau (2018).
